# Bacteriophage therapy as an alternative biocontrol against emerging multidrug resistant *E. coli* in broilers

**DOI:** 10.1016/j.sjbs.2022.02.015

**Published:** 2022-02-17

**Authors:** Samah Eid, Hala M.N. Tolba, Rehab I. Hamed, Nayera M. Al-Atfeehy

**Affiliations:** a Department of Bacteriology, Reference Laboratory for Veterinary Quality Control on Poultry Production (RLQP), Animal Health Research Institute (AHRI), Agriculture Research Center (ARC), P.O. Box 264-Dokki, Nadi El-Seidst., Giza 12618, Egypt; b Department of Avian and Rabbit Medicine, Faculty of Veterinary Medicine, Zagazig University, Zagazig 44519, Egypt; c Department of Poultry Diseases, Reference Laboratory for Veterinary Quality Control on Poultry Production (RLQP)-Sharkia Laboratory, Animal Health Research Institute (AHRI), Agricultural Research Centre (ARC), Egypt

**Keywords:** APEC, Bacteriophage, Escherichia *coli*, Antibiotic resistance, Biofilm, Phage therapy

## Abstract

Avian pathogenic *Escherichia coli* (APEC) is considered a severe issue to both poultry business and health of the general public. In that context, 50 samples from 250 diseased broiler chickens in 10 chicken farms were employed to *Escherichia coli* isolation. Microbiological techniques were employed to detect isolates of *E. coli* from 250 diseased broiler chickens which were examined by antimicrobial susceptibility profiles against 11 antimicrobial agents using disc diffusion technique as well as their biofilm forming capacity were detected. In addition to, study the isolation and purification of phages based on spot technique to verify that lytic phages are present in *E. coli* isolates and plaque assay for titration of bacteriophages. In the present research, we also looked at the ability of bacteriophages to inhibit and dissolve previously formed biofilms by *E. coli* O78 isolate. Moreover, experimental testing of *E. coli* O78 bacteriophages for colibacillosis prevention and control in one day old broiler chicks were done. The obtained results showed that twenty-six *E. coli* isolates out of 50 examined samples were isolated (10.4%). The most prevalent serotypes were O78, O121:H7, O146:H2, O124, O113:H4, O112:H2, O1:H7, O55:H7, O2:H6, O91:H21, O26:*H*11. Antibiogram results demonstrated the resistance of *E. coli* isolates with high percentage 100% were against, Ampicillin, Amoxicillin and Tetracycline. Biofilm quantification analysis showed that 24/26 (92.3%) isolates were considered biofilm producer isolates. The characterization and the lytic activity of bacteriophage were performed based on Transmission electron microscopy and showed the greatest lytic activity against the evaluated host strains with effective activity at concentration of 10^7^ at 24 h and strong significant reduction of the established *E. coli* O 78 biofilm within 12 h. The result of experimental infection showed that the performance indicators of phage in treated and challenged group showed high significant increase in body weight, weight gain and improved FCR than infected –antibiotic treated and infected bacteriophage and antibiotic treated. Total viable cell counts of *E. coli* in the lungs of birds revealed that there is highly significant difference between the six groups count results. We concluded that phage therapy found to be an attractive option to prevent and control multidrug resistant colibacillosis in broilers.

## 1 Introduction

Avian Colibacillosis is one of the most important avian bacterial diseases producing a wide spectrum of diseases in baby chicks, broilers, and layers leading to financial losses as well as a high rate of mortality and morbidity (Paixao et al., 2016). In broiler chickens, mortality varied from 20% to 40% depending on the disease severity and it most typically affects 3- to 12-week-old broiler chicks. *E. coli* is a typical component of the intestinal microbiota in chicken. The pathogenic *E. coli* strains are linked to extraintestinal illness resulting in high mortality and morbidity in poultry as a result of septicemia (Guabiraba and Schouler, 2015)**.** Also, APEC is the most common pathogen caused coli septicemia by commensal *E. coli* strains that lack virulence characteristics in stressed and immunocompromised birds. It also act as opportunistic secondary pathogens in the presence of respiratory diseases as Mycoplasmosis or infectious bronchitis diseases (Guabiraba and Schouler, 2015)**,** which characterized by high mortality rates with acute septicemia as well as airsacculitis, pericarditis, perihepatitis and peritonitis (Younis et al., 2017)**.** Antimicrobial drugs are unfortunately utilized not just for treatment, but also to improve animal productivity, growth rate and feed conversion rate in food-producing animals in many developing countries (Snary et al., 2004)*.* Many studies show that using antibiotics inappropriately to boost productivity increases the selection pressure for antimicrobial resistance bacteria (Levy, 2014). Antibiotic resistance shows no borders and bacteria that have gained resistance to specific antibiotics can quickly travel from nations with effective surveillance programs to those that do not. As a result, a coordinated strategy between developing countries is required (WHO, 2017). Antimicrobial resistance has the potential to wreak havoc on the treatment and management of infectious diseases in humans and animals alike. (Mamza et al., 2010). The application of an alternate rational approach is crucial in APEC control with non-antibiotic treatments. Probiotics, bacteriophages, growth inhibitors, virulence, innate immune stimulants and antimicrobial peptides have all been tested (Wang et al., 2017). A biofilm is a collection of microorganisms that form a community and are frequently found adhered to solid surfaces in damp environments. It is suspected that *Escherichia coli* is the main cause of many intestinal infections since infections are dependent on *E. coli* aggregation and biofilms are difficult to eliminate (Danese et al., 2000). Biofilm also makes it difficult for traditional antibiotics to penetrate the cells making them less responsive to medications (Mittal et al., 2015). As a result, there is a pressing need to investigate alternative treatment medicines to tackle infections caused by *E. coli* biofilm development isolates. The significant appearance of multidrug-resistant *E. coli* infections in chicken farms is obvious making treatment with standard antibiotics unfeasible and necessitating the use of alternative medicines. Phage therapy is one of the most effective ways to treat drug-resistant bacterial infections in animals without disrupting their normal gut flora**.** Bacteriophages have a number of qualities that make them potentially appealing therapeutic agents for bacterial illnesses. The excellent specificity and efficacy in lysing targeted harmful bacteria is one of them (Nabil et al., 2018)**.** Rydman and Bamford (2002) explained the strategy adopted during the phage bacterium interaction which based on the degradation of extracellular polymeric substance present in the biofilm by polysaccharide de-polymerases production allowing the access of phage which encased bacterial cells and cause their lysis**.** So, the goal of this study was to assess the prevalence, biofilm-forming ability, antibiotic resistance profile of *E. coli* isolated from diseased broilers and study bacteriophage therapy as a one of the alternative biocontrol measures against poultry colibacillosis.

## 2 Materials and methods

### 2.1 Sampling

Fifty samples from 250 broiler birds suspected to be infected with *E. coli*. Samples of air sacs and lungs were collected at 10 poultry farms in two provinces (Sharika & Giza) in Egypt with a history of respiratory diseases were selected in this study. In each farm, 25 birds were examined and each 5 birds represent as one sample so we collected five pooled air sacs and lungs samples from 25 birds from each flock. All birds showed signs of respiratory distress, dullness, depression, eye and nasal discharge, ruffled feather, diarrhea and mortalities. From each set of examined birds pooled lung and air sac were collected and transferred for further bacteriological examination.

### 2.2 *E. coli* isolation and identification

*Escherichia coli* isolation was carried out according to Lee et al. (2009). Briefly the samples were cultured on MacConkey agar at 37 °C for 24 h. Gram staining was performed to select gram negative and small rod bacteria.Isolates then subculture onto blood agar and incubate at 37 °C for 24 h to harvest the pure culture. Standard biochemical tests for *E. coli* identification were oxidase, urea, Tripple sugar iron (TSI) and citrate tests were used in accordance with MacFaddin (2000). *E. coli* isolates were serotyped in Reference Laboratory for Veterinary Quality Control on Poultry Production (RLQP) according to Lee et al. (2009).

### 2.3 Antibiotic susceptibility test

*Escherichia coli* isolates were submitted to antimicrobial sensitivity tests using method of disc diffusion as described by (Humphries et al., 2021)**.** The test was applied against 11 of the commonly used chemotherapeutic agents (Oxoid) which belong to 5 different antibiotic categories Ampicillin (10 μg), Amoxicillin (30 μg), streptomycin (10 µg), trimethoprim (10 µg), Amikacin (30 μg), Tetracycline (30 μg), Doxycycline (30 μg), Nalidixic acid (30 μg), Ciprofloxacin (5 μg), Levofloxacin (5 μg) and Norfloxacin (30 μg).

### 2.4 Isolation, enrichment and titration of bacteriophage

#### 2.4.1 Bacteriophage isolation and purification

Ten litter samples from positively infected broiler farms previously examined for presence of *E. coli* were utilized for bacteriophage isolation that was specific for APEC O 78 were homogenized in 20 ml homogenization buffer (0.25MKH_2_PO_4_ adjust to pH 7.2 with NaOH then centrifuged at 1200 rpm 14oc/10 min) and the supernatant was passed into an Eppendorf tube containing chloroform according to (Rahaman et al., 2014). Purification of phages was performed according to (Huang et al., 2018).

#### 2.4.2 1. Enrichment of the selected *E. coli* O78

The selected *E. coli* O78 isolate for bacteriophage assay based on demonstrated multidrug resistance ability and typical strong biofilm production. The prepared litter samples were added to the cultures of previously isolated bacteria during the mid-exponential growth phase and incubated at 12 h/37 °C.

#### 2.4.3 Spot technique

This test it was done to detect the presence of lytic phages in the prepared enriched phage filtrates (EP). Briefly, 10 μL of enriched phage filtrate which prepared from cultured *E. coli* O78 isolate on nutrient agar plates was spotted on the agar then incubated at 37 °C overnight for the detection clearance zones as lytic spots on the agar plates as by (Rahaman et al., 2014).

#### 2.4.4 Plaque assay

This assay was used for bacteriophages titration as described by (Akhtar et al., 2014)

#### 2.4.5 Transmission electron microscopy (TEM)

TEM was used to examine phages at the National Research Center (DOKKI, GIZA). According to (Fauquet et al., 2005) based on their morphology, phages were identified and classified.

### 2.5 In-vitro biofilm forming capacity of *Escherichia coli*

#### 2.5.1 Biofilm growth inhibition activities

Biofilm growth inhibition activity was used to test *E. coli* biofilm growth as reported by (Stepanović et al., 2000). Briefly, in triplicate wells, 20 μL of overnight *E. coli* suspension (10^6^ CFU/mL)for each isolate were put into microplate well. Microplates were aerobically incubated for 24 h, 48 h and 72 h at 37°Celsius. The broth was the only thing in the negative control wells. The contents of the plate were drained off and washed three times with sterile distilled water after incubation. Methanol was used to fix the wells, which were then air dried. Crystal violet was used to stain the microplates for 5 min. *E. coli* biofilm was measured based on the optical density (O.D.) of each well using a TECAN sunrise Microplate Reader tool at a wavelength of 570 nm. Strains were divided into four categories based on their OD values; no biofilm producers, weak, moderate, and strong biofilm producers.

#### 2.5.2 Biofilm degradation activity

The biofilm degradation activity was carried out in accordance with the procedure outlined by (Gharieb et al., 2020). After a 48-hour period, the bacteria were removed from the biofilm by washing with running water three times then the wells were filled with 200 ml of bacteriophage culture (10^8^ PFU/ml) and incubated for 48 h at 37 °C. Each microplate was stained for 15 min at room temperature with 200 ml of 1 percent crystal violet in each well. As a negative control, a microplate well containing bacterial suspension without the addition of bacteriophage was used. The microplate was washed according to the preventive, inhibitory treatments and the optical density (OD) of each treatment was measured using a TECAN sunrise Microplate ELISA reader.

### 2.6 Experimental application of *E. coli* O78 bacteriophage in one –day broiler chicks

#### 2.6.1 Ethical approval**a

The Ethical Committee of the Animal Health Research Institute (AHRI), ARC, Ministry of Agriculture, Giza, Egypt according to (OIE, 2021).

#### 2.6.2 Experimental design:

A total of 90 commercial one-day-old broiler chicks were raised from a local hatchery. Chicks were separated into six equal groups at random (15 birds per group) and given unlimited access to drinking water and starter meal. Chicks of group (1) were not treated and intratracheally (IT) challenged with *E. coli* 10^8^ CFU/ml (Tawakol et al., 2019). Group (2) was kept as non– infected non– treated as negative control. Group (3) birds were not infected but administered intratracheally with bacteriophage 10^8^PFU (Tawakol et al., 2019), Group (4) birds intratracheally administered bacteriophage 10^8^ PFU and intratracheal challenged with *E. coli*10^8^ CFU**.** Group (5) birds were challenged with E. *coli*10^8^ CFU and treated with antibiotic after appearance of clinical signs. Finally, group (6) was treated with both bacteriophage10^8^PFU and antibiotic after infection with E. *coli* 10^8^ CFU the infection was done at one day of age. In groups 4&6 the birds were treated with bacteriophage and then immediately challenged with *E. coli* (Huff et al., 2013)*.*

Bacterial strain selected multidrug resistant and strong biofilm producer E. *coli* O78 with concentration10^8 CFU^/ml. Bacteriophage 10^8^PFU/ml of isolated bacteriophage belongs to family Siphoviridae. Antibiotics doxycycline antibiotic powder: Doxycycline 20% (Pharma swede company)dose: 1 g \liter for 12 hrs for 5 successive days (Akbar et al., 2009). The clinical symptoms appeared post infection and number of dead bird were recorded, three birds from each group were collected at the time pointed (7 days, 15 days and 21 days post infection) for calculation of body weight, body weight gain and feed conversion was calculated as (Wagner et al., 1983). Moreover, lung samples were collected aseptically to enumerate *E. coli*, decimal dilutions of lung samples at the time pointed (7 days, 15 days and 21 days post infection) were plated onto MacConkey agar (Oxoid) by using a standard method (Smith and Huggins, 1982).

### 2.7 Statistical analysis

The data in this study were statistically analyzed by one-way A NOVA (Tamhane and Dunlop, 2000) using the MSTAT-C computer program. Results are presented as mean ± SE, and the statistical significance was set at (*p* ≤ 0.05). The significance between groups represented by small letters and the highest value represented by (a) letter.

## 3 Results

### 3.1 Signs and post mortem examination

The clinical findings of the suspected farms showed respiratory distress, dullness, depression, eye and nasal discharge, ruffled feather, diarrhea and mortalities with post mortem examination revealed congestion of all internal organ, pericarditis, perihepatitis and airsacculitis.

### 3.2 The cultural, biochemical and serotyping results of isolated *E. coli*

The study showed that 10.4% (26/50) broiler chicken samples in Sharika and Giza governorates were positive for *E. coli* isolation and biochemical identification as in (Table 1). *E. coli* serotypes were O78(5/26) 19.2%, O121: H7(4/26)15.4%, O146: H2(3/26) 11.5%, O124(3/26) 11.5%, O113: H4(2/26) 7.7%, O112: H2 (2/26) 7.7%, O1: H7 (2/26) 7.7%, O55: H7(2/26) 7.7%, O2: H6(1/26) 3.8%, O91: H21(1/26) 3.8%, O26: *H*11(1/26)3.8% (Table2).

**Table 1:** Incidence of *E. coli* isolated from broiler chicken farms in Sharkia and Giza governorates.

Governorate	No of farms	No of samples	No of positive isolates	Percent of positive isolates
Sharkia	6	150	15	6.00
Giza	4	100	11	4.4
Total	10	250	26	10.4

**Table 2:** Number of *E. coli* serotypes isolated from chicken farms.

Serotypes	Number of isolates	Rate of isolation
O78	5	19.2%*
O121: H7	4	15.4%
O146: H2	3	11.5%
O124	3	11.5%
O113: H4	2	7.7%
O112: H2	2	7.7%
O1: H7	2	7.7%
O55: H7	2	7.7%
O2: H6	1	3.8%
O91: H21	1	3.8%
O26: H11	1	3.8%
total	26	100%

* The percentage was calculated according to the number of positive samples.

### 3.3 Prevalence of antimicrobial resistance

The isolated *E. coli* tested against 11 different antimicrobial agents from 5 different classes of antimicrobial agents as in (Table3). The result showed that the highest resistance rate (100%) was demonstrated against Ampicillin, Amoxicillin and Tetracycline followed by Amikacin (76.9%), Norfloxacin (73.1%), streptomycin (69.2%), Trimethoprim and Nalidexic acid (65.4%), Ciprofloxacin (53.8%), Doxycycline and Levofloxacin (50%). Multiple resistances were detected in 100% of the isolates.

**Table 3:** Anti-microbial susceptibility profile of 26 *E. coli* isolates.

Antibiotic Group	Antibiotic	Disc Potency Mg/disc	Number of Resistant Strains (%)
Aminoglycosides	Streptomycin	10 µg	18(69.2%)
	Amikacin	30 µg	20(76.9%)
Tetracyclines	Tetracycline	30 µg	26(100%)
	Doxycycline	30 µg	13(50%)
Quinolones	Nalidexic acid	30 μg	17 (65.4%)
	Norfloxacin	10ug	19 (73.1%)
	Ciprofloxacin	5 μg	14 (53.8%)
	Levofloxacin	5 μg	13(50%)
β-Lactams	Ampicillin	10ug	26(100%)
	Amoxicillin	30 μg	26(100%)
Diaminopyrimidine	Trimethoprim	10 μg	17 (65.4%)

### 3.4 Bacteriophage isolation, enrichment, and titration

**Fig. 1:**
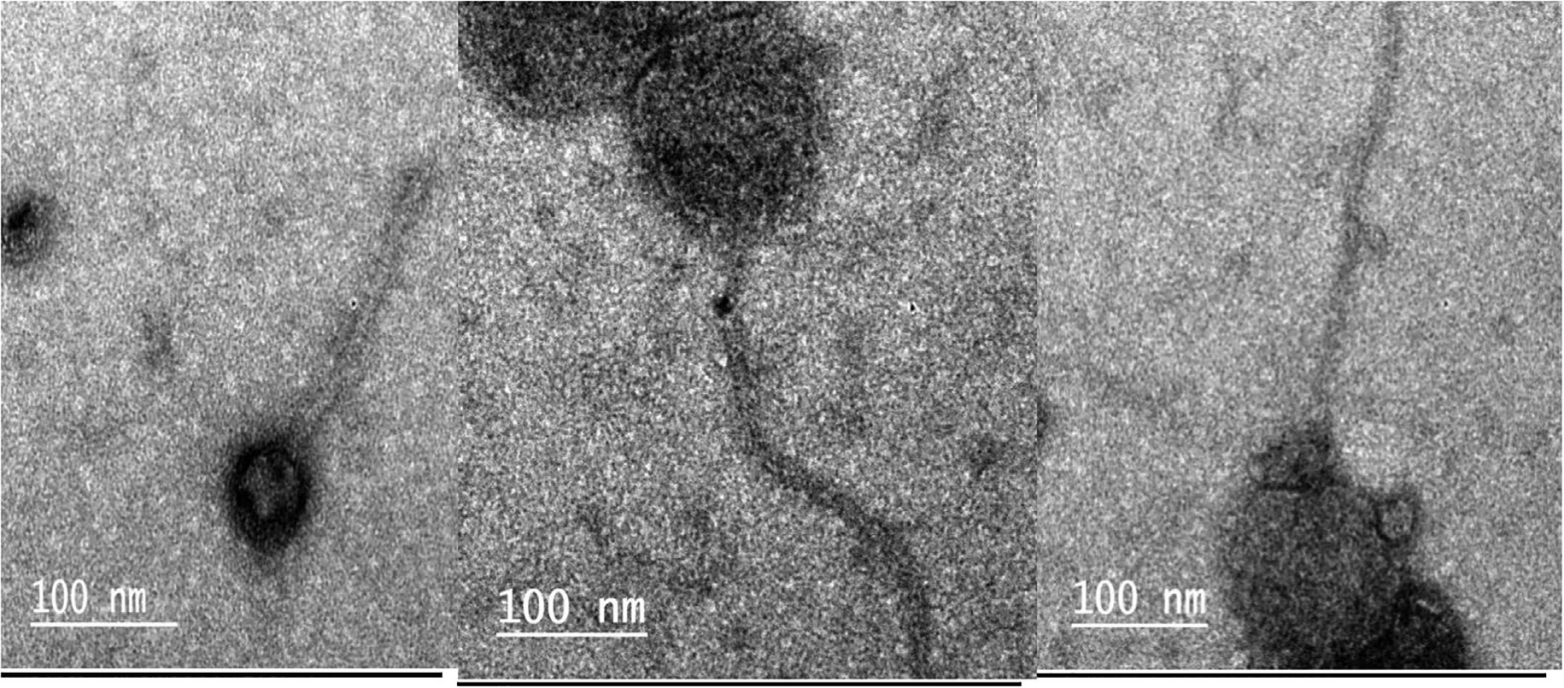
Electron micrograph of phages of the E *coli* O 78, all of them related to Siphoviridae family which have long noncontractile tail stained with 2% w/v uranyl acetate.

**Fig. 2:**
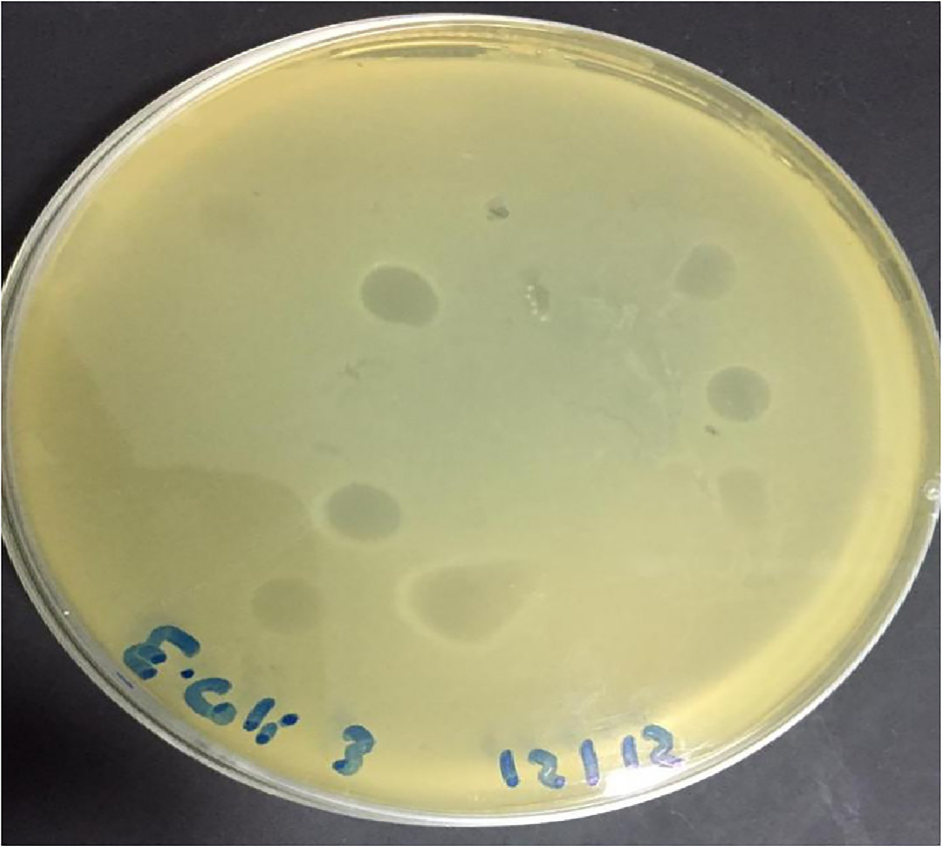

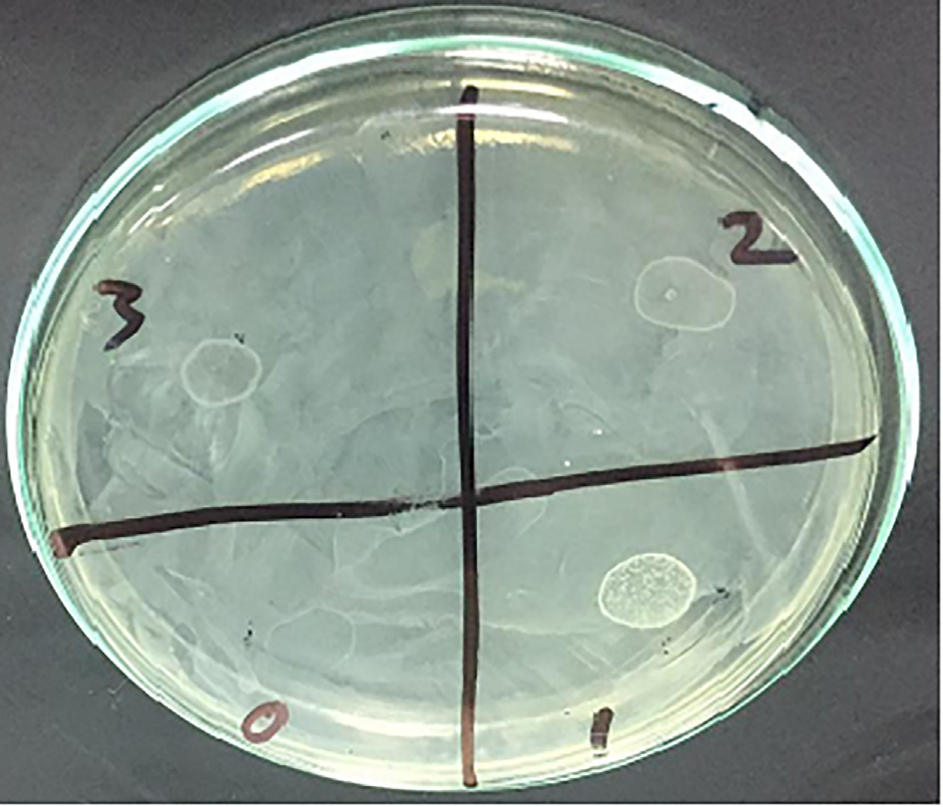
**a.** spot technique showed Large clear phage plaque. **2b**. spot technique showed control negative (saline only) (0), lysogenic phages (1,2,3).

**Fig. 3:**
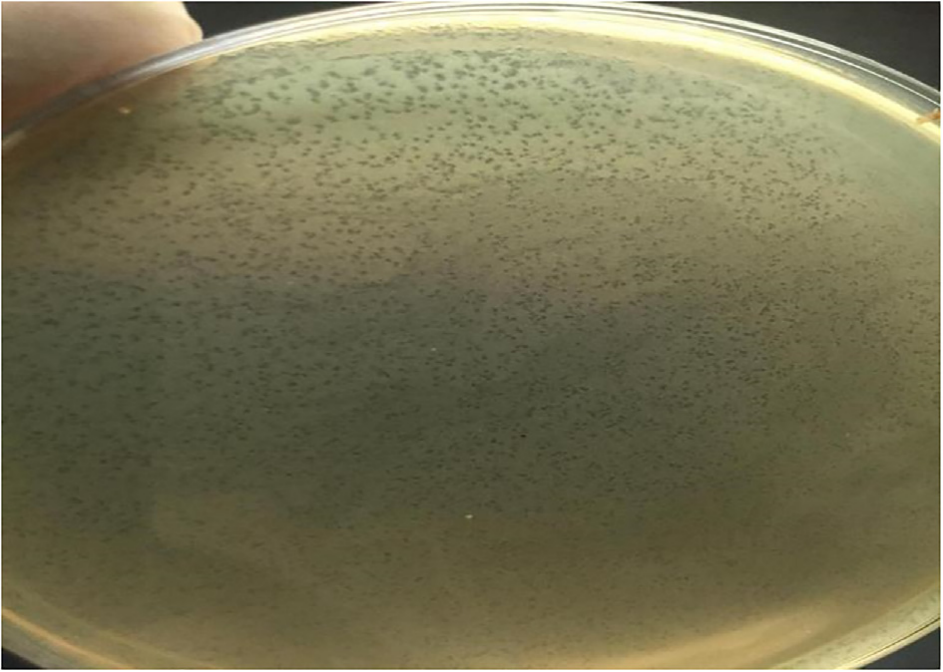

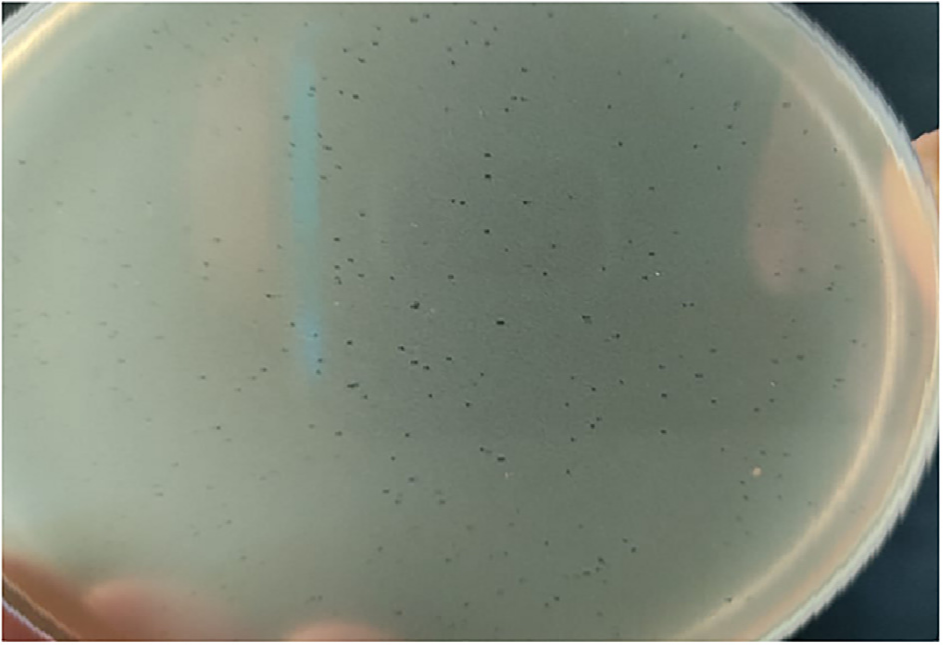

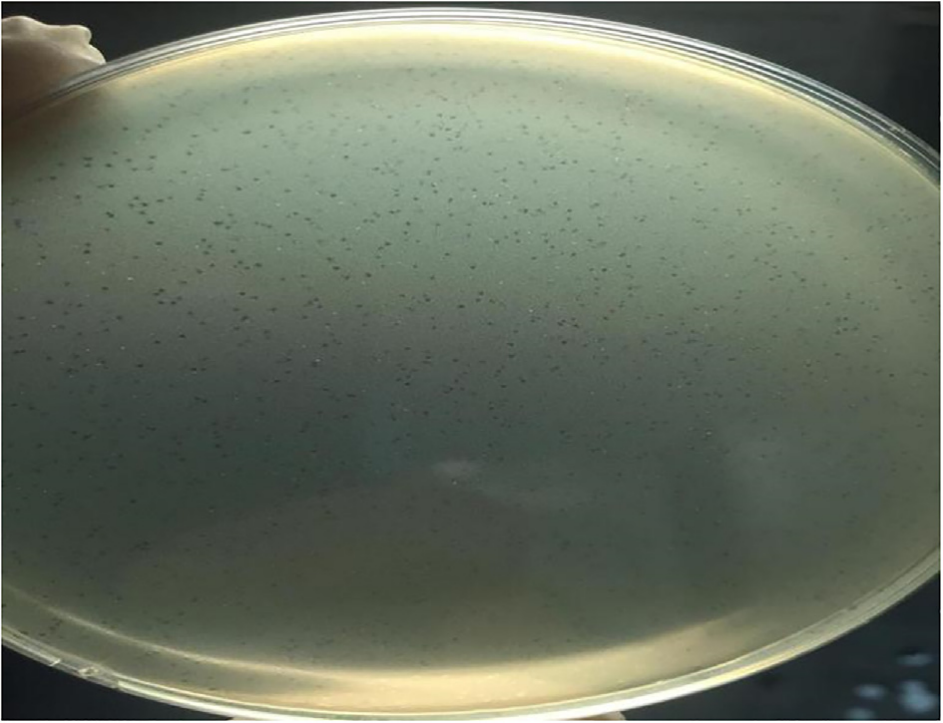

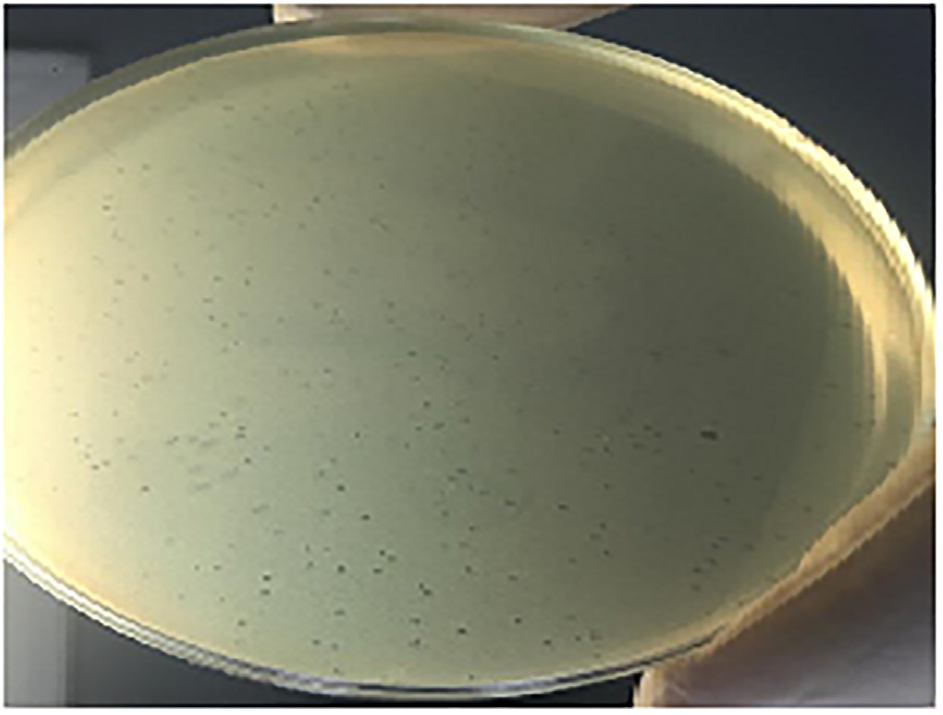
**a**. Tiny lytic areas (plaque formation) on plaque assay method. **3b**. small-sized countable clear plaque on plaque assay method. **3c**. small-sized pin-headed, clear plaque on plaque assay method. 3d. small-sized countable clear plaque on plaque assay method.

Three lytic phages were isolated from three of the ten (3/10) broiler litter samples according to spot and plague assays (Fig. 2 and Fig. 3). Based on TEM micrograph, phage was morphologically like λ phages that belongs to family Siphoviridae which has icosahedral head with a diameter size of 647 nm and long thin non-contractile tail of 125 nm (Fig. 1).

### 3.5 Quantification of biofilm

Biofilm quantification analysis showed that 92.3% (24\26) of isolates were biofilm producers with the following categories of biofilm production: 7.7% of isolates (2\26) were non-adherent (non-biofilm producer) (-), 19.2% of isolates (5\26) were weakly adherent (+), 30.8% of isolates (8\26) were moderately adherent (++), and 42.3% of isolates (11\26) were strongly adherent (+++) as shown in (Table 4).

**Table 4:** Phenotypic biofilm production profile of the tested 26 *E. coli* isolates.

Biofilm profile	No. of isolates (%)
Non-biofilm producer (−)	2 /26 (7.7%)
Weak biofilm producer (+)	5/26 (19.2%)
Moderate biofilm producer (++)	8 /26 (30.8%)
Strong biofilm producer (+++)	11/26 (42.3%)
Total biofilm producer	24/26 (92.3%)

**Table 5:** Effect of lytic phage and doxycycline on body performance in non-infected and experimentally infected chicken with *E. coli* O78.

Groups	Body performance (1 to 7 days)			Body performance (7 to 15 days)			Body performance (15to 21 days)		
	BW (g)	BW gain (g)	FCR	BW (g)	BW gain (g)	FCR	BW (g)	BW gain (g)	FCR
Group (1)	151.6 ± 2.8c	107.3 ± 2.31c	1.31 ± 0.03a	294.6 ± 8.39c	146.0 ± 9.8c	1.94 ± 0.03a	488 ± 7.55d	193.3 ± 14.9c	1.95 ± 0.03a
Group (2)	180.0 ± 2.0a	136.6 ± 2.31a	0.96 ± 0.02c	467.3 ± 24.1a	287.3 ± 22.1a	1.50 ± 0.02d	782 ± 24.6a	315.3 ± 21.7a	1.54 ± 0.03d
Group (3)	180.3 ± 2.2a	136.0 ± 2.65a	0.96 ± 0.02c	468.3 ± 20.1a	287.6 ± 19.7a	1.48 ± 0.03d	781 ± 7.6a	313.3 ± 27.5a	1.55 ± 0.03d
Group (4)	170.0 ± 2.0b	125.6 ± 2.08b	1.07 ± 0.03b	370.0 ± 13.3d	198.0 ± 11.3d	1.67 ± 0.07c	637 ± 7.55b	285.0 ± 8.19d	1.62 ± 0.03c
Group (5)	169.0 ± 1.0b	124.6 ± 1.15b	1.08 ± 0.01b	341.0 ± 2.65b	172.0 ± 3.61b	1.78 ± 0.02b	606 ± 7.64c	265.6 ± 5.51b	1.75 ± 0.02b
Group (6)	170.3 ± 2.5b	126.3 ± 2.52b	1.07 ± 0.03b	353.0 ± 6.08b	182.6 ± 6.66b	1.75 ± 0.03b	615 ± 5.1c	267. ± 10.5b	1.72 ± 0.01b

Data are means (n = 3) ± SE. The same letters in each column indicate not significant differences according to the LSD test (P ≤ 0.05). BW; Body weight, BW gain; Body weight gain, FCR; Food conversion rate.

**Table 6:** Effect of lytic phage and doxycycline on total viable cell counts of *E. coli* in the lung of experimentally infected chicken.

Groups date	Infected non treated group	Group infected and treated with bacteriophage	Group infected and treated with doxycycline	Group infected and treated with bacteriophage and doxycycline
At 7 days	5.63 ± 0.02	1.93 ± 0.02	1.99 ± 0.02	1.55 ± 0.02
At 15 days	5.95 ± 0.02	1.77 ± 0.02	1.82 ± 0.02	1.55 ± 0.02
At 21 days	6.31 ± 0.02	1.63 ± 0.02	1.67 ± 0.02	1.47 ± 0.02

### 3.6 Effect of lytic phage and doxycycline on body performance in healthy and experimentally infected chicken with *E. coli* O78

In the untreated challenged group 1, clinical indications occurred 48 to 72 h after infection in the form of dullness, depression, drooping of wings, not eating, ruffled feathers, inability to stand, gasping and sneezing. By the third day after infection, there had been a number of deaths (4 out of 15 birds). Postmortem examination of dead and sacrificed birds revealed liver and lung congestion, severe pericarditis and perihepatitis. When birds were given bacteriophage and challenged with *E. coli,* they showed minimal symptoms compared to the positive control group (1) with no mortality noted. Also, it revealed a range of conditions including normal appearance, minor lung congestion and mild inflammation in the intestines as in group (6) challenged and treated with both antibiotic and bacteriophage. In group 5 challenged and treated only with antibiotic clinical signs were more severe than bacteriophage challenged group slight perihepatitis and pericarditis was found, mortality recorded at 5 days post infection (1 out 15).

There was significant reduction in B.W, B.W gain and FCR in the positive control group (1) (488.00 ± 7.55, 193.33 ± 14.98 and 1.95 ± 0.03) at the age 15–21 days as compared with negative control group (2) and group (3) administrated phage only (782.67 ± 24.68, 315.33 ± 21.78 and 1.54 ± 0.03), (781.67 ± 7.64, 313.33 ± 27.54 and 1.55 ± 0.03) respectively (Table 5). The treated groups (4, 5 and 6) showed significant improvement in body weight (BW), body weight gain (BWG) and feed conversion rate (FCR) were (637.00 ± 7.55, 280.00 ± 8.19 and 1.62 ± 0.03), (606.67 ± 7.64, 265.67 ± 5.51 and 1.75 ± 0.02) and (615.00 ± 5.00 ,267.00 ± 10.58 and 1.72 ± 0.01) respectively as compared with infected group. meanwhile the group (4) showed significant improvement than group (5 and 6). *E. coli* bacterial counts in lungs were collected at 7-, 15- and 21-day post infection. The results showed that challenged and non-treated group showed a significantly higher APEC, which increased gradually at 15- and 21-day post infection. On other side, group treated antibiotic and groups treated with bacteriophage alone or with antibiotic showed significantly reduced APEC comparing with infected non treated group. Meanwhile the group treated with both bacteriophage and antibiotic showed significantly reduce APEC comparing with group treated with bacteriophage alone or group treated with antibiotic only (Table 6).

### 3.7 Prevention of *E. coli* biofilm by lytic phage

**Fig. 4:**
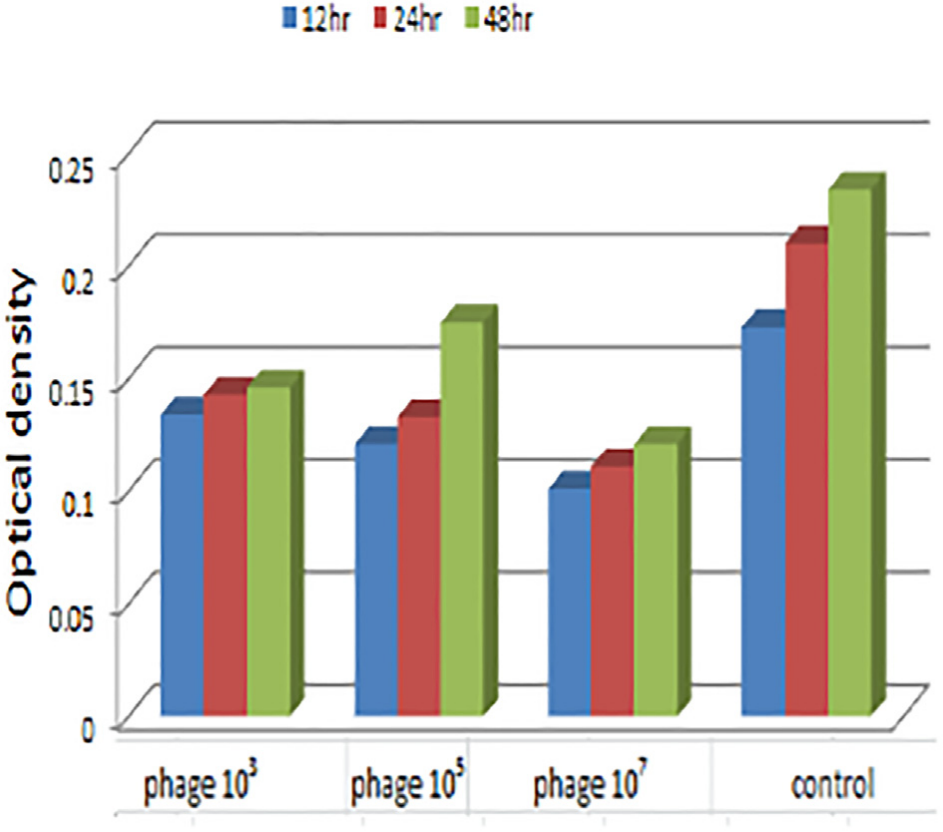
Biofilm growth inhibition activities in different concentrations of 10^3^, 10^5^ and 10^7^*E. coli* O78 phage compared with control among 24 h, 48 h, and 72 h.

**Fig. 5:**
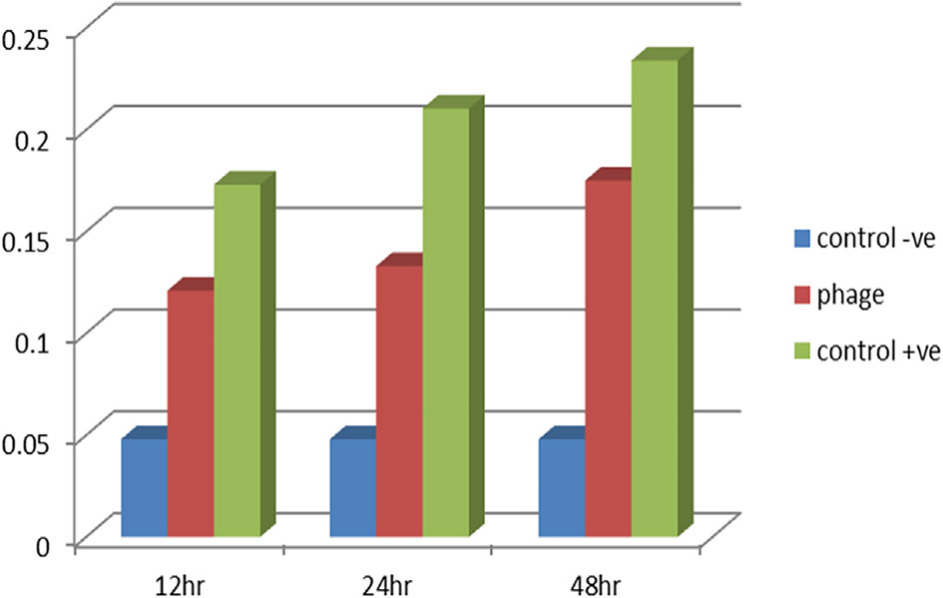
The reduction of established *E. coli* O 78 biofilm by treating with phage compared with controls at 37 °C.

Optical density demonstrated a higher activity of phage concentration 10^7^ at 24 h followed by the same concentration at 48 h and 72 h in inhibiting biofilm formation in phage treated isolates compared to control as in (Fig. 4 and Table 7). There was highly significant difference between different groups at *p* ≤ 0.05. With comparing to the untreated control, the phage reduced the developed *E. coli* O78 biofilm biomass. (Fig. 5 and Table 8). The significant reduction in biofilm showed strongly at 12 h compared with the controls.

**Table 7:** One-way a nova analysis of the optical density results of Biofilm growth inhibition activities in different concentrations of 10^3^, 10^5^ and 10^7^*E coli* phage compared with control among 24 h, 48 h, and 72 h.

	Sum of Squares	df	Mean Square	F	Sig.
Between Groups	0.014	3	0.005	0.066	0.004
Within Groups	0.004	8	0.000		
Total	0.018	11

**Table 8:** One-way a nova analysis of the optical density results of the reduction of established *E. coli* O 78 biofilm by treating with phage compared with controls at 37 °C.

	Sum of Squares	df	Mean Square	F	Sig.
Between Groups	0.038	2	0.019	32.462	0.001
Within Groups	0.004	6	0.001		
Total	0.041	8			

## 4 Discussion

Avian Pathogenic *Escherichia coli* (APEC) is one of the most real problems facing the poultry industry. *E. coli* isolated from diseased birds were identified based on morphological, cultural and biochemical characteristics. Interestingly, a total of 26 farms with percentage of 10.4% were found to be positive for *E. coli* from 250 diseased and freshly dead samples (colibacillosis suspected) in Sharkia and Giza governorates. The *E. coli* incidence *in* the present study was in close to (Radwan et al., 2021). Differences in *E. coli* prevalence rates in broilers might be credited with different management approaches to sanitary and environmental conditions in chicken farms such as overcrowding, inadequate ventilation and elevated air ammonia levels or differences in microbiological quality such as *E. coli* count in water, litter, feed and air or due to strain pathogenicity, virulence differences and finally due to bird immunological status (Abd El Tawab et al., 2015). Moreover, this variation thought to be a result of the seasons as they recorded *E. coli* higher prevalence rate was reported in winter (60.9%) than that in summer (41%) according to (Abd El Tawab et al., 2015)**.** Antibiotics are being used more frequently in broiler chickens for treatment and as a growth promoter was also blamed for the prevalent heterogeneity of *E. coli* as reported by (White et al., 2001) and (Nwankwo et al., 2014).

Although several APEC serotypes were linked to colibacillosis, three serotypes (O78, O2, and O1) account for more than 80% of outbreaks (Ghunaim et al., 2014). The current results showed eleven different serotypes among the total 26 isolates (Table 2), of which O78 had highest detection rate (5/26) 19.2% among the total 26 isolates. Kathayat et al. (2021) declared that serogroups O1, O2 and O18 of APEC isolates could be a source of human extraintestinal infections because they share virulence genes and genetic similarities with human uropathogenic *E. coli* (UPEC), neonatal meningitis *E. coli* (NMEC) causing urinary tract infections and meningitis in humans. Antibiotic misuse in veterinary medicine, industry and agriculture has been blamed. The relevance of the strong relationship between humans and animals as well as the environment, is reaffirmed by one health strategy regarding the direct transmission of resistant bacteria from humans to animals and vice versa as well through waste from the poultry farm to the environment. It was obviously noted that all the isolated *E. coli* serotypes in the current study showed multidrug resistance against three/or more antimicrobials of different categories. In this study, all the isolated *E. coli* showed 100% resistance rate against Ampicillin, Amoxicillin and Tetracycline followed by Amikacin (76.9%), Norfloxacin (73.1%), Streptomycin (69.2%), Trimethoprim (65.4%), Nalidexic acid (65.4%), Ciprofloxacin (53.8%), Doxycycline (50%), Levofloxacin (50%). The resistance considered as a consequence of indiscriminate use of antimicrobials. These mechanisms causing the bacteria to adopt one of three changes within the existing genome mutations , proteome (phase variation) of a bacteria or by the formation of bacterial cell interactions (mixed bacterial biofilms) and horizontal transfer of new genes (acquisition) (Kuznetsova et al., 2020)**.** Moreover, (Volkova et al., 2014) thought that transduction by bacteriophages has a modest role to play in these horizontal antimicrobial resistance gene transfer which is thought to occur as a result of conjugative plasmids, transposons or naked DNA uptake because of the global rise of numerous drug-resistant bacteria, antibiotics that were considered as an “endangering species” so the world has increased new interest to bacteriophages as promising antimicrobial agents (Dalmasso et al., 2016).

Bacterial biofilm development is frequently regarded as a pathogenicity factor (Musk Jr and Hergenrother, 2006). However, a closer examination reveals that the biofilm phenotype also protects cells from phage attack. It typically confers antibiotic tolerance by protecting cells from antibiotics not just by establishing a physical barrier but also because the innermost cells are less metabolically active and hence less impacted by antibiotics (Fauconnier, 2017, Stewart, 2015). The investigation of biofilm production showed that 24 out of 26 (92.3%) of the isolates were biofilm producers. Gonçalves et al. (2017) elucidated the link between biofilm development and antibiotic resistance resulting in drugs having difficulties penetrating bacterial cells that secrete a polymeric matrix containing polysaccharide, proteins and DNA. As a result, alternative novel treatments aimed at lowering antibiotic use are required and bacteriophages could be a promising option. Coliphages are bacteriophages that infect *E. coli*. Bacteriophages come in a wide variety of shapes and sizes and can be classified according to their genome, morphology and host specificity (Schwarzer et al., 2012), Because new phages are discovered every day and the International Committee on Taxonomy of Viruses (ICTV) is behind on its classification timetable, phage classification is an open-ended process (Ackermann, 2009)*.* The number of Bacteriophages of recognized morphology is over 5,500 (Ackermann, 2007)*.* Bacteriophages are distinguished by their morphology which is why transmission electron microscopy (TEM) is still necessary (Ackermann, 2012). In the current investigation TEM was applied to classify and morphologically examine them and the results revealed that phages were morphologically like λ phages (family: Siphoviridae) which is characterized by an icosahedral head and long thin non-contractile tail (Fig. 1). Phages can be lytic or nonlytic resulting in death of host bacterial cells in the former case and the generation of bacteria with phage DNA integrated into their genomes in the latter instance. In general, phages can be virulent (lytic) or temperate (lysogenic); certain families have carrier states. Although both lytic and lysogenic phages are found in the environment and only lytic phages are employed in phage therapy because lysogenic phages integrate their DNA into the bacteria and hence are unable to destroy it (Karthik et al., 2014). As a model organism *E. coli* has been employed in a variety of biological engineering and industrial microbiology investigations (Lee, 1996). Biofilm matrix density, low metabolic state of biofilm cells and rapid proliferation of phage resistant variants were thought to have an impact on biofilm and phage interactions. Sulakvelidze et al. (2001) determined the beneficial effect of the interaction of bacteriophage with bacterial biofilms in the prevention of biofilms formation. Statistical analysis of the present study revealed highly a substantial distinction between groupings at *p* ≤ 0.05 and the highest preventive activity of the isolated phages at concentration of 10^7^ within 24 h against biofilm formation by phage treated *E*. *coli* O78 isolates followed by the same concentration at 48 h and 72 h respectively compared with control (Fig. 4 and Table7). Moreover, the current results revealed that phage has the capacity to reduce the established *E. coli* O78 biofilm biomass (Fig. 5 and Table 8), the significant reduction in biofilm within 12 h compared with controls. In experiments, phages were found to be capable of lysing host bacteria in biofilms made from single and mixed bacterial species. Sutherland et al. (2004) reported that phage therapy assumed as an attractive option to prevent and control the formation of biofilm related infections Verma et al. (2010) concluded that the biofilms eradication requires the combination of both antibiotics and bacteriophages, while (Hughes et al., 1998) hypothesized that combining bacteriophages with DNase enzymes efficiently destroys the biofilm matrix. However, Pallavali et al. (2019) demonstrated that the biofilm may be removed purely by the bacteriophage itself and deemed it a significant development in the hunt for additional agents to replace antibiotics were consistent with the findings of the current investigation. Biofilm makes it difficult for traditional antibiotics to penetrate the cells making them less responsive to antibiotics (Mittal et al., 2015). According to Schwarzer et al. (2012) bacteriophages are divided into groups based on their genome, morphology and host specificity. While some bacteriophages are host specific, others are broad-host-range and able to invade different bacterial species (Evans et al., 2010). Sulakvelidze et al. (2001) reported the beneficial preventive biofilm formation effect resulted from the interaction of bacteriophage with bacterial biofilms. Anti-biofilm activity test in vitro including Biofilm growth inhibition and degradation activity showed a higher activity of phage of concentration at 10^7^ for 24 h followed by the same concentration for 48 h and 72 h respectively, in phage-treated isolates to avoid biofilm development these results compatible with the theory of Pei and Lamas-Samanamud (2014) who concluded that phages in industrial and clinical fields act as antibiofilm agents including phage therapy, filtration membranes and biofilm infected medical devices. The current results also revealed that the phage reduced the established *E. coli* O78 biofilm biomass .The significant reduction in biofilm showed strongly at 12 h compared with the controls. Sagar et al. (2015) reported that a lytic P2 bacteriophage's impact on several biofilm stages (initial biofilm formation, matrix establishment and biofilm maturation) produced by Pseudomonas aeruginosa has been hypothesized as well as its potential to kill bacterial cells during the early stage of biofilm development. During the phage bacterium interaction phages create polysaccharide depolymerases to breakdown extracellular polymeric material present in the biofilm allowing phages to reach encapsulated bacterial cells and cause lysis (Rydman and Bamford, 2002)**.** The experimentally infected chicks with. *E. coli* and not treated group (1) recorded mortalities (26.7%) this result was in agreement with Sorour et al. (2020) who found that mortality rate 30%. Also infected group with *E. coli* and treated with doxycycline group (5) was resulted in mortality to (6.66%) that matched with Akbar et al. (2009) who reported that doxycycline reduces mortality rate from 76% to 56 %. On other side no mortalities associated with other challenged and treated groups (4 and 6) this finding were supported with (Lau et al., 2010). Tawakol et al., 2019, Sorour et al., 2020 whom recorded no mortalities were found after treatment with bacteriophage intratracheal (IT), Huff et al. (2013) has been established that the a etiology of colibacillosis is a pulmonary infection that swiftly progresses to a systemic illness. Complete protection was observed when bacteriophage and *E. coli* were both given Intratracheal (IT) but other routes such as coarse and fine spray were unable to deliver sufficient titers of bacteriophage to the trachea to prevent the onset of colibacillosis as mortalities were reported by (Huff et al., 2013) when challenged birds and treated with coarse spray of bacteriophage more over mortalities were recorded Intramuscular preparations have also been tried by (Barrow et al., 1998) against *E. coli* infection in chickens and shown promising results in the treatment of septicemia therefore we found that high protection by the bacteriophage application with higher dose of the phage, at a titer of 10^8^ PFU, and intratracheal route gave perfect protection to the birds against the development of infection with *E. coli* matched with the dose and route of administration this matched with (Huff et al., 2002). (Coulter et al., 2014) recorded that remarkable decrease in antibiotic-resistant *E. coli* by the combination of phages and antibiotic, the results demonstrated that using bacteriophage in combination with doxycycline has effect of synergy improving treatments for colibacillosis effectiveness. With regard to performance indicators of Phage treated and challenged group (4) we found that significant increase in body weight, weight gain and improvement in FCR (637,00 ± 7.55b &280.00 ± 819d and 1.62 ± 03c) when compared with infected –antibiotic treated group (5) (606 ± 67c, 265.67 ± 5.51b, 1.75 ± 0.02) and group challenged and treated with both bacteriophage and antibiotic group (6) (615,00 ± 5.00c, 276.00 ± 10..58b, 1.72 ± 0.01b). This is may be because of the phage's strong specificity as phage do not affect other profitable microbes so able to keep a favorable intestinal micro-ecological balance, un like the antibiotic that may affect the beneficial bacteria this findings supported by (Xie et al., 2005) who found that group treated with phage showed better body weight and FCR than antibiotic treated group so our findings revealed a number of advantages to phages suggesting that phages should be used for the prevention and treatment of colibacillosis instead of antibiotics. On the other hand, comparing the performance parameters of the infected untreated group to the negative group and the group administered phage only revealed that the infected untreated group had a low significant level weekly body weight, feed consumption, body weight gain, and bad FCR. These findings were consistent with (Salim et al., 2013). Pallavali et al. (2019) suggested that decrease in weight gain due to oxidative stress caused by colibacillosis. Of interest we found that a higher bacteriophage dose and intra-tracheal inoculation lead to significantly decrease shedding of *E. coli* in lung tissue comparing with infected non treated group (Tawakol et al., 2019). In addition to there was no significant difference between the treatment group by antibiotic or by phage in lung tissue counts (P ≤ 0.05) while The largest reduction of this count was measured on bird group (6) challenged and treated with both antibiotic and bacteriophage these results are in close agreement with (Wernicki et al., 2017) who proposed that a combination of enrofloxacin and bacteriophage treatments could be more effective and useful in colibacillosis control.

## 5 Conclusion

Our results concluded that bacteriophage is useful alternative to antibiotics specially for preventing and treating the multidrug resistance infections. It is also concluded that combination between the antibiotic and bacteriophage therapy reduce and rationalize the levels of antibiotics used in treating bacterial diseases. Finally, future investigations recommended to focus mechanisms of phage-bacterium interactions and application of phage -based products intended for treatment colibacillosis in poultry farms.

## Declaration of Competing Interest

The authors declare that they have no known competing financial interests or personal relationships that could have appeared to influence the work reported in this paper.
